# Robot-assisted radical prostatectomy in a patient with Zinner syndrome

**DOI:** 10.1016/j.ijscr.2025.110895

**Published:** 2025-01-15

**Authors:** Wojciech Narożański, Mateusz Glembin, Dawid Romanowicz

**Affiliations:** Department of Urology, St. Adalbert's Hospital, COPERNICUS Healthcare Entity Ltd., Gdańsk, Poland

**Keywords:** Zinner syndrome, Prostate cancer, Robotic surgery, Radical prostatectomy, Da Vinci system

## Abstract

**Introduction and importance:**

To our knowledge, this is the first reported case of Robot-Assisted Radical Prostatectomy (RARP) in a patient with Zinner syndrome, a rare congenital condition characterized by unilateral renal agenesis, seminal vesicle cysts, and ejaculatory duct obstruction.

**Case presentation:**

A 64-year-old male with elevated PSA was diagnosed with low-risk prostate cancer. Preoperative imaging confirmed Zinner syndrome, posing unique anatomical challenges. The procedure was successfully performed using the Da Vinci robotic system, allowing precise removal of the prostate, non-functioning kidney, and dilated ureter from the standard port setting without changing the patient's position. The surgery was completed with minimal complications, and the patient recovered well.

**Clinical discussion:**

The anatomical anomalies associated with Zinner syndrome complicate standard surgical approaches. Robotic-assisted techniques provide enhanced visualization and precision, facilitating safe and effective management of these cases.

**Conclusion:**

This urological procedure can be safely and efficiently performed on patients with Zinner Syndrome and synchronous prostate cancer by urological surgeons with extensive experience in robotic surgery in high-volume centers.

## Highlights

• First reported case of Robot-Assisted Radical Prostatectomy in Zinner syndrome
• Successful simultaneous removal of prostate, non-functioning kidney, and dilated ureter without changing patient positioning
• Robotic-assisted approaches for patients with Zinner syndrome and prostate cancer
• Safety and efficacy of robotic surgery in complex congenital urological cases

## 1 Introduction

Zinner syndrome, first described in 1914 [1], is a rare congenital anomaly involving unilateral renal agenesis, seminal vesicle cysts, and ejaculatory duct obstruction. It often presents in adulthood with urinary symptoms or pelvic pain and is rarely associated with malignancies such as prostate cancer. Due to the limited occurrence of Zinner syndrome, the co-occurrence with prostate cancer is poorly documented. This case report describes the first known Robot-Assisted Radical Prostatectomy (RARP) performed simultaneously with nephroureterectomy in a patient with Zinner syndrome, emphasizing the challenges posed by the patient's unique anatomy and the efficacy of robotic surgery in such cases.

## 2 Case presentation

A 64-year-old male, previously asymptomatic, presented for a routine urological evaluation with elevated PSA levels (6.35 ng/mL). A multiparametric MRI revealed multiple focal lesions in the transitional zone of the prostate, with PI-RADS 4 characteristics. Lesions included a 9 mm bilateral base segment and a 6 mm right-sided mid-prostate lesion. Digital rectal examination showed increased density, but no evident nodular abnormalities.

Subsequent fusion biopsy revealed benign prostatic hyperplasia with focal high-grade prostatic intraepithelial neoplasia (PIN HG). Mapping cores from the right lobe confirmed acinar adenocarcinoma (Gleason 3 + 3, Grade Group 1) in 4 out of 6 cores, with no evidence of neurovascular invasion or extraprostatic extension. The left lobe showed benign prostatic hyperplasia and glandular atrophy. MRI also revealed a cyst of the right seminal vesicle, with associated protrusion into the mesorectum.

The patient was presented with all available treatment options for low-risk prostate cancer, including active surveillance, low-dose brachytherapy, and external beam radiation therapy EBRT without ADT. After a detailed discussion of the risks, benefits, and potential outcomes of each approach, the patient made an informed decision to pursue surgical treatment with radical prostatectomy.

Further diagnostic evaluation including CT scans, revealed an aplastic non-contrast excreting right kidney with a dilated ureter and hypertrophy of the left kidney (with proper nephrogram). The right ureter, enlarged and filled with dense content, was in direct contact with the prostate and cystically enlarged seminal vesicles. These imaging findings fit the criteria for Zinner syndrome, as described in the imaging report. Typically, the ectopic ureter drains into the urinary bladder; however, in this case, it drained into the prostatic urethra, which necessitated its removal.

Bone scintigraphy showed no evidence of metastasis.

The non-functional status of the right kidney was determined based on preoperative imaging. Contrast-enhanced computed tomography described the kidney as aplastic, non-contrast excreting with no discernible renal parenchyma or functional cortical structures. Additional functional studies, such as a renogram were not performed, as the imaging findings and surgical context indicated that the kidney was non-functional. We did not perform additional tests on the ejaculatory ducts as it would not have affected the treatment we proposed.

The patient was staged as T1cN0M0 low risk prostate cancer and was scheduled for radical prostatectomy using the Da Vinci X robotic system, with concurrent removal of the aplastic kidney and ureter.

Preoperatively, the patient received standard anticoagulant prophylaxis with enoxaparin (40 mg) administered the evening before surgery, along with a single preoperative dose of cefuroxime (1500 mg) for infection prophylaxis.

**Fig. 1:**
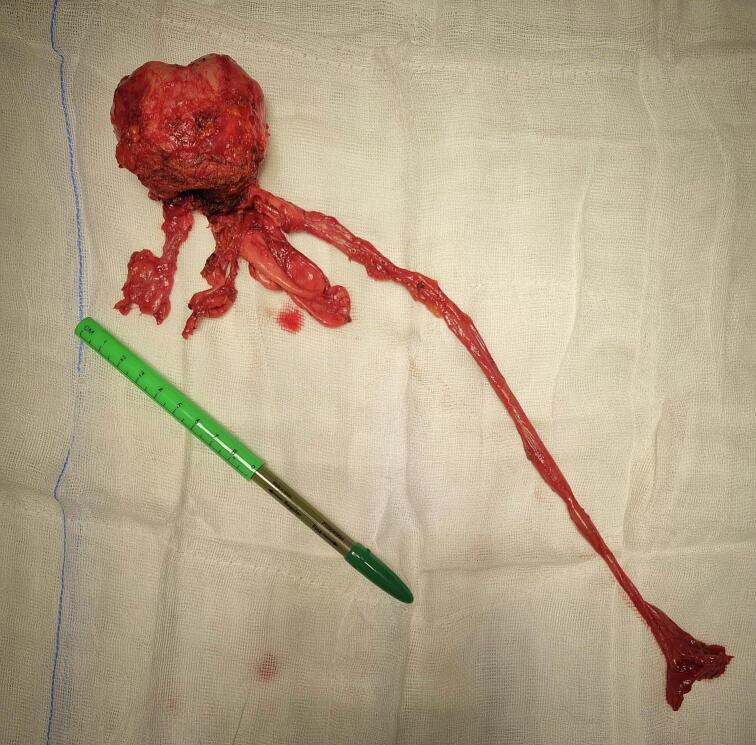
Speciment. A macroscopic view of the excised prostate along with the right seminal vesicle cyst, the dilated right ureter with the agenetic kidney.

The patient was placed on a standard vacuum mattress, positioned in the Trendelenburg position at a 29-degree angle. A rectal drain was inserted to monitor rectal integrity after prostate removal. Access to the abdominal cavity was achieved using microlaparotomy through an extended 5 cm incision, which was also used for specimen extraction at the end of the procedure [Fig. 1]. Three sutures were placed on the abdominal fascia to facilitate easy closure after surgery.

Four robotic trocars were inserted transperitoneally, with trocar number 4 placed on the right side of the abdomen. Additionally, two assistance ports were positioned: a 5 mm port located 7 cm to the left and below the leftmost robotic trocar, and a 12 mm port placed above the left anterior superior iliac spine.

**Fig. 2:**
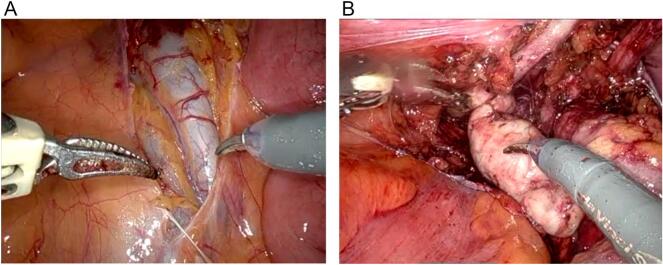
a b First stage of operation.

**Fig. 3:**
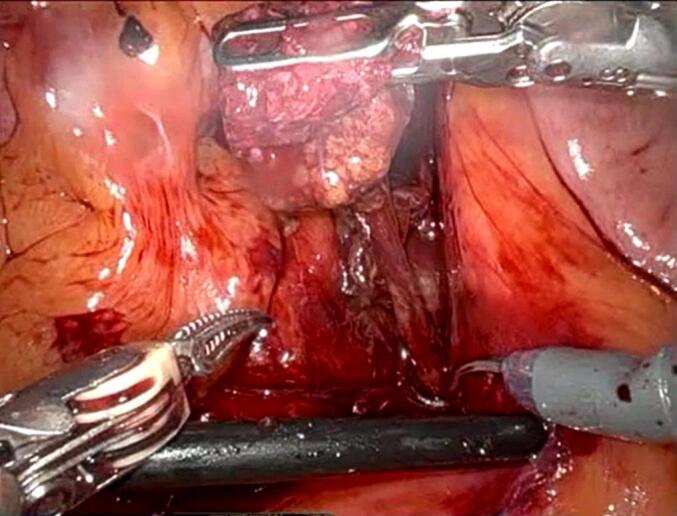
Second stage of operation.

The procedure began with an incision of the peritoneum above the rectal pouch to expose and dissect both vas deferens and seminal vesicles [Fig. 2a]. On the right side, a large cyst of the right seminal vesicle and a dilated right ureter were identified [Fig. 2b]. The right ureter was found to be draining directly into the prostatic urethra. Careful dissection of the right ureter was carried out, and it was traced proximally to the non-functioning aplastic kidney. During the dissection of the kidney, a brownish fluid was observed, which showed no bacterial growth in postoperative testing. A nephrectomy was performed, with the right kidney excised en bloc with its renal pedicle [Fig. 3]. Two hemoclips were applied to secure the renal pedicle before it was transected.

The prostate was then separated from the bladder, with the bladder neck preservation. An extrafascial dissection of the prostate was performed, during which neurovascular bundles were secured with Hem-o-lok clips to minimize bleeding and preserve as much nerve function as possible. The urethra was divided, and the prostate was removed.

It's worth noting that we didn't need to change the trocar placement to remove the aplastic left kidney along with the ureter.

A Rocco suture was used for posterior reconstruction of the bladder neck, a standard procedure in our center. This was followed by a single-layer continuous Van Velthoven anastomosis using Stratafix 3–0 sutures, with bilateral anterior suspension applied to secure the anastomosis. An 18F silicone catheter was inserted to drain the bladder, and a 16F Redon drain was placed to prevent fluid accumulation in the surgical site.

The total operative time was 3 h and 20 min, with an estimated blood loss of 100 mL. Postoperatively, the drain was removed on the first day due to minimal fluid accumulation, and the patient was started on a soft diet. The patient was discharged from the hospital on the second postoperative day without early complications. The urinary catheter was removed on the tenth postoperative day.

## 3 Results

Histopathological examination revealed carcinoma in approximately 5 % of the surface area of the right lobe, with positive surgical margins at the right prostatic urethra. No evidence of microscopic extraprostatic extension was noted. The final pathological result was pT2 N0 M0, Gleason 4 + 3 (Grade Group 3), with an R1 margin. The first postoperative PSA level after 8 weeks was 0.006 ng/mL. The patient reported partial erectile function recovery with tadalafil 5 mg daily and mild urinary incontinence (lasted up to 4 weeks after the procedure). After pelvic floor muscle strengthening exercises the patient only uses the security pad.

Postoperative histopathological examination revealed moreover fragments of fibrous-adipose tissue containing vascular and neural cross-sections. Prominent fibrotic structures with calcifications, cystic spaces lined by flattened cuboidal epithelium, and tubular formations were observed. These findings are consistent with the diagnosis of an aplastic kidney. The ureter resection margin was of normal histological structure.

The patient's eGFR was 88.8 mL/min/1.73 m^2^ preoperatively and 86.6 mL/min/1.73 m^2^ postoperatively. This minimal change in renal function demonstrates the compensatory ability of the contralateral kidney and the absence of significant renal impairment after surgery.

## 4 Discussion

To our knowledge, this is the first reported case of Robot-Assisted Radical Prostatectomy (RARP) with simultaneous nephroureterectomy in a patient with Zinner's syndrome. The syndrome itself is rare, the combination of Zinner's syndrome and prostate cancer is even more unusual, making this case particularly significant for both diagnostic and therapeutic considerations.

A review of the literature reveals very few cases where patients with Zinner's syndrome required radical prostatectomy. Most of the available reports focus on the management of the congenital syndrome itself rather than concurrent prostate cancer. In fact, only Spinos et al. [2] described a case of laparoscopic radical prostatectomy (LRP) performed in a patient with Zinner's syndrome. Their approach involved the use of 3D laparoscopy, which enabled precise dissection and the safe management of pelvic structures. However, the challenges of such a complex anatomical presentation, particularly the presence of a dilated ureter and seminal vesicle cyst, make the utilization of robotic assistance even more advantageous.

In our case, the Da Vinci robotic system offered superior precision and visualization compared to traditional laparoscopic approaches. The system's flexibility allowed us to navigate the patient's complex anatomy, including the presence of the aplastic kidney and the dilated ureter, without the need for patient repositioning or re-docking of the robotic arms. This stands in contrast to the experience of Kiremit et al. [3], who encountered difficulties with the robotic arms and camera positioning, which necessitated changing the patient's position to a lateral decubitus during the procedure. Moreover, Deameyer et al. [4] described a case in which they opted not to perform a nephrectomy alongside vesiculectomy due to concerns about the complexity of the patient's anatomical presentation and the potential need for patient repositioning. In our case, we successfully completed both the nephrectomy and prostatectomy in a single session without changing the patient's position. The removal of the non-functioning kidney and dilated ureter in our case was particularly important to alleviate potential complications from the malformed urinary tract structures.

This case not only demonstrates the capability of robotic-assisted surgery in handling complex urological cases but also highlights the importance of individualized surgical planning.

From an oncological perspective, the final histopathology in our case revealed a pT2, N0, R1 prostate cancer, with cancerous tissue found at the surgical margin of the right-sided prostatic urethra. The patient's postoperative PSA levels after 8 weeks were low (0.006 ng/mL), suggesting successful oncological control in the short term. However, the positive surgical margin raises concerns about potential recurrence, which will need to be closely monitored during follow-up.

Overall, our experience supports the notion that robotic-assisted surgery offers significant advantages in managing complex cases involving congenital anomalies and malignancies. High-volume centers with experienced surgeons can safely and efficiently perform such complicated cases due to their familiarity with advanced techniques and equipment. Future studies and additional case reports will help build a more robust understanding of the optimal management strategies for patients with Zinner's syndrome and concomitant conditions like prostate cancer.

## 5 Conclusion

This case highlights the potential and safety of robotic-assisted surgery in managing prostate cancer complicated by congenital anomalies such as Zinner syndrome. Future studies and case reports will further explore the potential of robotic surgery in similarly challenging scenarios.

## Author contribution

Wojciech Narożański performed the surgical operation. Mateusz Glembin and Dawid Romanowicz assisted in the preparation of the manuscript and contributed to the creation of the video documentation of the surgery, ensuring a comprehensive presentation and analysis of the procedure. All authors contributed to the writing process, reviewed, and approved the final manuscript.

## Consent

Written informed consent was obtained from the patient for publication and any accompanying images. A copy of the written consent is available for review by the Editor-in-Chief of this journal on request.

## Ethical approval

Ethical approval was not required for this case report, as the described procedure was a standard clinical operation performed in accordance with established medical guidelines and did not involve any experimental intervention or research beyond routine care. According to the regulations of the Bioethics Committee in Gdańsk, which is responsible for assessing the ethical permissibility of medical experiments based on ethical criteria, as well as the feasibility and purpose of such research, this case report does not meet the criteria requiring ethical review. Additionally, the case report is based on a retrospective review of the patient's clinical data, and all necessary precautions have been taken to ensure patient confidentiality and privacy. The patient has provided informed consent for the publication of this case and the associated data.

## Guarantor

Wojciech Narożański, MD, PhD.

Mateusz Glembin, MD 1.

Dawid Romanowicz, MD 1.

## Research registration number

Not applicable.

## Methods

The work has been reported in line with the SCARE criteria [5].

## Declaration of Generative AI and AI-assisted technologies in the writing process

During the preparation of this work, the authors used Generative AI and AI-assisted technologies (chatGPT) only to check grammar and language review.

## Funding

This research did not receive any specific grant from funding agencies in the public, commercial, or not-for-profit sectors.

## Conflict of interest statement

The authors declare no conflict of interest.

## Data Availability

The datasets generated or analysed during the current study are available from the corresponding author on reasonable request.

## Appendix A Supplementary data

Supplementary material Image 1
