# Supplementary material for: Robot-assisted radical prostatectomy in a patient with Zinner syndrome
Source: Int J Surg Case Rep. 2025 Jan 15;128:110895. doi: 10.1016/j.ijscr.2025.110895 (PMC11848090; doi:10.1016/j.ijscr.2025.110895)
Supplement: Supplementary file 1 — Supplementary material [file mmc1.docx]

**Supplementary video**

<https://drive.google.com/file/d/1B6bXhVaXn-52zhLLutiHrKhSZAOueaaV/view?usp=drive_link>


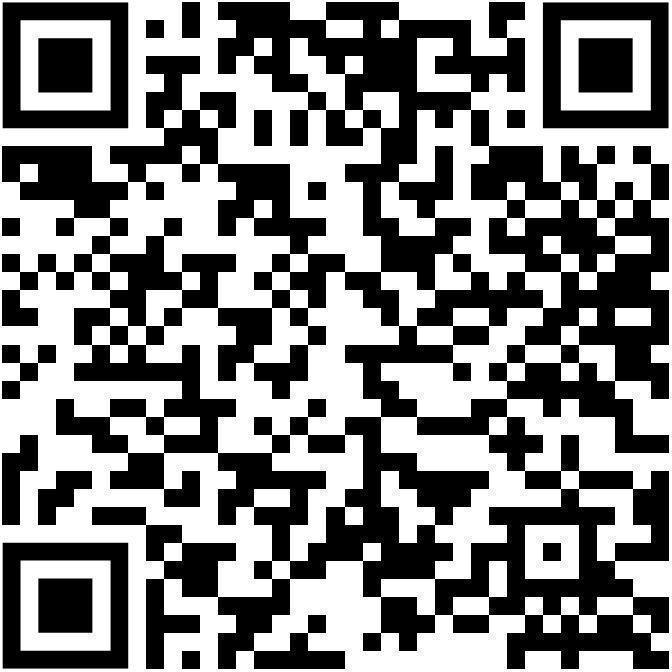


- Legend
  - 00:00 Visualization of the ureter
  - 06:45 Visualization of the vas deferens
  - 09:55 Visualization of the follicular cyst
  - 15:45 View of the follicle, follicular cyst, and vas deferens
  - 15:55-49:00 Removal of the aplastic kidney
  - 49:00 The vas deferens is pulled from the prostate side, with the right ureter draining to the prostatic urethra. The dissected kidney and ureter are removed by pulling the tissues retrovesically
  - 1:05:20 Specimen excision
